# Assessing alexithymia in chronic pain: psychometric properties of the Toronto Alexithymia Scale-20 and Perth Alexithymia Questionnaire

**DOI:** 10.1097/PR9.0000000000001204

**Published:** 2024-12-09

**Authors:** Rachel V. Aaron, David A. Preece, Lauren C. Heathcote, Stephen T. Wegener, Claudia M. Campbell, Chung Jung Mun

**Affiliations:** a Department of Physical Medicine and Rehabilitation, Johns Hopkins School of Medicine, Baltimore, MD, USA; b School of Population Health, Curtin University, Perth, Australia; c School of Psychological Science, The University of Western Australia, Perth, Australia; d Health Psychology Section, Institute of Psychiatry Psychology and Neuroscience, Kings College London, London, United Kingdom; e Department of Psychiatry and Behavioral Sciences, Johns Hopkins School of Medicine, Baltimore, MD, USA; f Edson College of Nursing and Health Innovation, Arizona State University, Phoenix, AZ, USA

**Keywords:** Alexithymia, Psychometrics, Chronic pain, Emotional awareness, Validity

## Abstract

**Introduction:**

Alexithymia is elevated in chronic pain and relates to poor pain-related outcomes. However, despite concerns from other clinical populations, the psychometric properties of alexithymia measures have not been rigorously established in chronic pain.

**Objective:**

This study examined the psychometric properties of the Toronto Alexithymia Scale-20 Item (TAS-20) and the Perth Alexithymia Questionnaire (PAQ) in adults with chronic pain.

**Methods:**

An online sample of adults with chronic pain across the United States (N = 1453) completed the TAS-20, PAQ, and related questionnaires at baseline, 3-month follow-up, and 12-month follow-up.

**Results:**

Both measures showed good temporal stability, convergent validity (with emotion regulation scores), divergent validity (with depression and anxiety scores), and criterion validity. Some concerns were raised about the TAS-20: the original 3-factor structure showed a poor model fit; the Externally Oriented Thinking subscale of the TAS-20 had poor factor loadings and unacceptable internal consistency; and, we identified several TAS-20 items that may slightly inflate the predictive validity of the TAS-20 on pain-related outcomes. The original 5-factor structure of the PAQ showed a good fit; each PAQ subscale had good factor loadings and excellent internal consistency.

**Conclusions:**

Both the TAS-20 and PAQ had psychometric strengths. Our data raised some concern for the use of TAS-20 subscales; the PAQ may be a psychometrically stronger option, particularly for investigators interested in alexithymia subscale analysis in people with chronic pain.

## Abstract 2

Supplemental Digital Content is Available in the Text.

The Toronto Alexithymia Scale-20 Item and Perth Alexithymia Questionnaire had psychometric strengths for assessing alexithymia in chronic pain; several weaknesses of the Toronto Alexithymia Scale-20 Item were identified.

## 1. Introduction

Chronic pain occurs in a third of adults^37^ and is associated with functional impairment and elevated psychological distress.^4,37,44^ *Alexithymia*, meaning “no words for emotions” in Greek, was originally coined to refer to a trait comprised of difficulties in emotional awareness and a tendency toward operational thinking.^45,54^ Most modern alexithymia assessment tools measure 3 emotion processing deficits, including difficulties identifying one's feelings (DIF), difficulties describing feelings (DDF), and externally oriented thinking (EOT).^41,54^ Dozens of studies show that alexithymia is elevated in chronic pain and relates to greater pain intensity, physical interference, depression, and anxiety.^1^ However, to our knowledge, there have not been studies dedicated to investigating the psychometric properties of any available alexithymia measures in people with chronic pain. The current study tested the psychometric properties of the 20-item Toronto Alexithymia Scale (TAS-20)^5^ and the Perth Alexithymia Questionnaire (PAQ).^38^

The TAS-20 (comprised of DIF, DDF, and EOT subscales) has been widely used in people with chronic pain.^1^ The TAS-20 generally demonstrates good reliability and validity across a range of sample types.^6^ The TAS-20 total scores and DIF and DDF subscales consistently demonstrate acceptable to high reliability, and the factor structure is typically broadly consistent with the intended 3-factor (DIF, DDF, EOT) model.^50^ Some concerns relate to the EOT subscale, which often has unacceptably low reliability (α < 0.70) and several items with low factor loadings on their intended factor, which can reduce the model fit below acceptable cutoffs in some samples.^8,18^ The original developers caution against interpreting TAS-20 subscales and recommend to focus principally on the total score.^8^

To our knowledge, the content validity of TAS-20 has not been investigated in chronic pain; however, 3 somatic-focused items could potentially inflate the severity of alexithymia scores among people with chronic pain.^53^ For example, the item “I have physical sensations that even doctors don't understand” describes a common experience among people with primary pain conditions (ie, potentially inflating convergence between TAS-20 scores and pain-related outcomes). The results of 1 confirmatory factor analysis (CFA) showed that 2 somatic-focused TAS-20 items formed a new latent factor focused on interoceptive ability.^15^ Other factor analytic examinations have highlighted that these items may overlap with somatic markers of distress.^30,40^ The inclusion of somatic items in psychological inventories could lead to false positives among people with chronic pain due to poor content validity,^51^ and it is possible that similar findings could emerge in the assessment of alexithymia if somatic items are included. This requires dedicated empirical testing.

The PAQ was developed to assess domains of alexithymia (DIF, DDF, EOT) at the total score and subscale level. Designed to enable valence-specific assessments,^20^ the PAQ assesses DIF and DDF for both positive and negative affect.^38^ Parsing negative and positive affect may have utility as positive and negative emotions serve distinct functions,^16^ and positive affect is a common target of chronic pain intervention.^33,47^ Perth Alexithymia Questionnaire items were developed to refer specifically to emotions rather than bodily sensations,^38^ which could minimize concern that items within reflect somatic symptoms that confound with the experience of pain. Although the PAQ is relatively new, a growing number of psychometric studies have been published, suggesting good reliability^38,39^ and validity^11,29^ at the total score and subscale levels, and an intended 5 factor (subscale) structure that replicates in community and clinical samples.^17,39^ To our knowledge, the PAQ has not been tested in people with chronic pain.

Comparative studies in nonclinical samples suggest that the TAS-20 and PAQ can both perform well and correlate highly with one another. A primary difference is the lower reliability for the TAS-20 EOT score.^39^ There is some debate about whether the PAQ's capacity to extract facet-level (subscale) and valence-specific scores adds incremental validity to the TAS-20, with some arguing that the TAS-20 total score is sufficient for alexithymia assessments^54^ and others arguing that different alexithymia facets and valence domains can contribute significant unique variance to the prediction of clinical outcomes.^43^

The current study aimed to determine the psychometric properties of the TAS-20 and PAQ in adults with chronic pain. We tested the measures in original 3-factor structure of the TAS-20 and 5-factor structure of the PAQ, reliability, convergent validity, divergent validity, and predictive validity for pain-related constructs.

## 2. Methods

These data were collected as part of a prospective online study of adults with chronic pain across the United States focused on emotion regulation^2,3^ and the impact of coronavirus disease 2019 on chronic pain.^34,35^ The aims of the current paper are distinct from prior publications.

### 2.1. Participants

Participants were adults with mixed chronic pain conditions. At baseline, oversampling was used to achieve a sample that was demographically representative of the broader chronic pain population in the United States with respect to gender, race, and ethnicity. The procedures are described in greater detail elsewhere.^35^ We collected data using Amazon Mechanical Turk at 3 time points beginning in 2020: baseline (T1), 3-month follow-up (T2), and 12-month follow-up (T3). Eligibility criteria included (1) 18 or older, (2) average pain severity of ≥3/10, (3) reside in the United States, (4) proficient in English language, and (5) willing to participate in follow-up assessment. Only participants with an Amazon Mechanical Turk approval rating of 95% or higher were invited to participate. Participants had to pass 3/3 embedded attention checks and achieve other quality indicators.^35^ All participants who were included in T1 (N = 1453) were invited to complete T2 (N = 884; 61% retention) and T3 (N = 813; 56% retention). A total of 610 participants had data available at all 3 time points.

### 2.2. Measures

#### 2.2.1. Demographic questionnaire

Participants reported age, gender, race, ethnicity, education, employment, income, and marital status.

#### 2.2.2. Toronto Alexithymia Scale 20-Item

The TAS-20, a 20-item self-report questionnaire,^5^ includes subscales: (1) DIF (eg, “I am often confused about what emotion I am feeling.”), (2) DDF (eg, “It is difficult for me to find the right words for my feelings”), and (3) EOT (“Being in touch with emotions is essential” [reverse scored]). Toronto Alexithymia Scale-20 Item total and subscale scores are created by summing responses. Participants report the extent to which they agree with items on a 5-point Likert scale. Higher scores indicate higher alexithymia.

#### 2.2.3. Perth Alexithymia Questionnaire

The PAQ^38^ is a 24-item self-report measure of alexithymia that assesses 5 alexithymia subscales, including negative and positive emotions: (1) Negative-DIF (eg, “When I'm feeling *bad*, I can't make sense of those feelings.”), (2) Positive-DIF (eg, “When I'm feeling *good*, I can't make sense of those feelings.”), (3) Negative-DDF (eg,” When something *bad* happens, it's hard for me to put into words how I'm feeling.”), (4) Positive-DDF (eg, “When something *good* happens, it's hard for me to put into words how I'm feeling.”), and (5) General-EOT (“I tend to ignore how I feel.”). Subscales can be summed into composite scores, including a total scale score. Items are answered on a 7-point Likert scale. Higher scores indicate higher alexithymia.

#### 2.2.4. Brief Pain Inventory: Short Form

Participants rated their current, worst, least, and average pain intensity in the past 24 hours, using a 0 to 10 Likert scale.^12^ Pain severity was calculated by averaging these 4 ratings. Participants reported the extent to which pain interfered with 7 domains (ie, general activity, mood, walking ability, normal work, relations with other people, sleep, and enjoyment of life) on a 0 to 10 Likert scale. Pain interference was calculated by averaging these 7 ratings. The Brief Pain Inventory has good psychometric properties.^24,48^ In the current study, internal consistency was excellent for pain severity (0.90-0.92) and pain interference (0.92-0.93).

#### 2.2.5. Difficulties in Emotion Regulation Scale 16-item

The 16-item Difficulties in Emotion Regulation Scale (DERS-16)^9^ is an abbreviated version of the 36-item DERS,^19^ which assesses emotion dysregulation. The current study used DERS-16 total score and the subscale “lack of emotional clarity,” which is includes 2 items (“I have difficulty making sense of my feelings,” “I am confused about how I feel”). Participants indicated the extent to which statements apply to them using a 5-point Likert scale. The DERS-16 has good psychometric properties.^9^ Internal consistency for the total score was 0.91 in the current study.

#### 2.2.6. Patient-Reported Outcomes Measurement Information System Depression and Anxiety Scales

The Patient-Reported Outcomes Measurement Information System (PROMIS)^10^ is a set of measures that assess various outcomes. In the current study, the Depression and Anxiety Short Forms (4a) were used, each comprised of 4 items. Participants indicated how often, over the past 7 days, they experienced symptoms on a 5-point Likert scale. As recommended, for each measure, total raw scores were summed and transformed into standardized T-scores calibrated against the general population, with a mean of 50 and a SD of ±10. The PROMIS measures have good psychometric properties in chronic pain.^27^ In the current study, internal consistency for anxiety was 0.90 and for depression was 0.92.

### 2.3. Data analytic strategy

Toronto Alexithymia Scale-20 Item data were available at T1, T2, and T3. The PAQ was recently developed^38^ and added to the survey battery at T2 after the investigators became aware of it. Thus, PAQ data were available at T2 and T3. When findings are presented descriptively or individually based (eg, descriptive statistics, temporal stability), data from all relevant time points are included. Otherwise, analyses consider T2 and T3 only, to facilitate comparison of the TAS-20 and PAQ.

#### 2.3.1. Descriptive statistics

We described demographic characteristics and TAS-20 and PAQ total and subscale scores at each time point and checked for group differences in TAS-20 and PAQ total scores using Pearson correlations (age) and 1-way analysis of variances (gender, race, education).

#### 2.3.2. Confirmatory factor analysis

**Figure 1.**
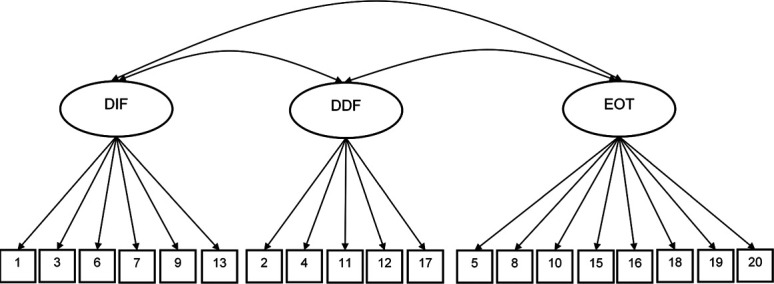
Toronto Alexithymia Scale 20-item confirmatory factor analysis model. DDF, difficulty describing feelings; DIF, difficulty identifying feelings; EOT, externally oriented thinking. Ellipses: latent factors. Squares: observed variables (item numbers). Each factor indicator had an error term but not shown here.

**Figure 2.**
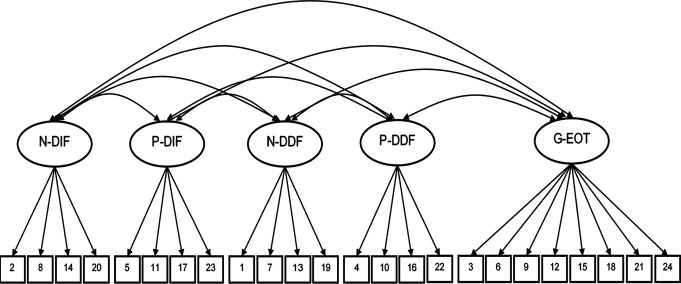
Perth Alexithymia Questionnaire confirmatory factor analysis model. DDF, difficulty describing feelings; DIF, difficulty identifying feelings; EOT, externally oriented thinking; G, general; N, negative; P, positive. Ellipses: latent factors. Squares: observed variables (item numbers). Each factor indicator had an error term but not shown here.

Confirmatory factor analysis was conducted using Mplus version 8^36^ to test the original 3-factor structure of the TAS-20 and the original 5-factor structure of the PAQ (Figs. 1 and 2). We focused on CFA of each scale's intended structure to inform previous findings, which have relied on the 3-factor structure of the TAS-20 in chronic pain. First, single factor models were fit as a parsimonious baseline to examine whether the TAS-20 and PAQ were measuring multidimensional constructs. Each model was evaluated using the recommended cutoff scores for acceptable model fit across a range of commonly used fit index values (ie, root mean square error of approximation [RMSEA] ≤0.08, comparative fit index [CFI] ≥0.90, and standardized root mean residual [SRMR] ≤0.08).^23^ Factor loadings ≥0.40 were considered meaningful loadings.^46^

#### 2.3.3. Reliability

##### 2.3.3.1. Internal consistency

Cronbach α and McDonald ω were calculated to evaluate the internal consistency of the TAS-20 and PAQ total and subscale scores. Values ≥0.70 were considered acceptable, ≥0.80 considered good, and ≥0.90 considered excellent for both α and ω.^21^

##### 2.3.3.2. Temporal stability

Pearson correlations were calculated between TAS-20 total and subscale scores and between PAQ total and subscale scores across time. Strong correlations between the same measure across time were interpreted to indicate good temporal stability.

#### 2.3.4. Construct validity

##### 2.3.4.1. Criterion validity

Criterion validity was assessed by evaluating Pearson correlations between TAS-20 total and subscales scores with PAQ total and subscale scores. There are no strict cutoffs to determine adequate construct validity.^14^ For the current study, it was determined that large correlations would be interpreted as adequate criterion validity. Benchmarks of small (*r* = 0.10-0.30), medium (*r* = 0.31-0.50), and large (*r* > 0.50) were applied for all analyses.

##### 2.3.4.2. Convergent and divergent validity

Pearson correlations were calculated between TAS-20 and PAQ total and subscale scores with DERS total and clarity subscale. Large correlations were interpreted as evidence of adequate convergent validity, as alexithymia has been shown to impair emotion regulation.^42^ Pearson correlations were calculated between TAS-20 and PAQ total and subscale scores with PROMIS Depression and Anxiety. Given known positive correlations between alexithymia and depression and anxiety, large correlations were interpreted as concerning for poor discriminant validity.

##### 2.3.4.3. Predictive validity

First, we conducted 4 path analyses to test associations between TAS-20 total and PAQ total at T2 on pain intensity and pain interference at T3 (while controlling for the dependent variables at T2). Next, to address the concern that associations between alexithymia and pain may be confounded by psychological distress in some samples, we conducted a sensitivity analysis controlling for depression and anxiety at T2. Finally, to address concerns that somatic-focused items may inflate the predictive validity of the TAS-20 on pain outcomes, we conducted a set of sensitivity analyses with the 3 somatic-focused items (3, 7, 13) removed from the TAS-20.

## 3. Results

### 3.1. Sample characteristics

Sample characteristics have been reported elsewhere^3^ but replicated here for reference (Table 1). Average pain duration was 10.9 years (SD = 9.1). Average pain severity was 4.2 (SD = 1.6), and average pain interference was 4.9 (SD = 2.4). The results of an attrition analysis have been reported elsewhere^35^; briefly, participants who were younger, male, Hispanic, and who had lower educational level were statistically more likely to be missing at 1-year follow-up. Pain-related experiences at baseline were not significantly associated with missingness.

**Table 1:** Sample characteristics at baseline.

Variables	Mean (SD) or n (%)
Age (y)	41.7 (13.1)
Gender	
Female	968 (65.2)
Male	498 (34.3)
Other (nonbinary/genderqueer)	7 (0.5)
Race	
White	1189 (81.9)
Black/African American	99 (6.8)
Asian/Asian American	50 (3.4)
American Indian/Alaska Native	13 (0.9)
Native Hawaiian/Pacific Islander	0 (0)
Mixed race	67 (4.6)
Other race	28 (1.9)
Prefer not to answer	5 (0.3)
Don't know	1 (0.1)
Ethnicity	
Hispanic	144 (9.9)
Non-Hispanic	1297 (89.4)
Prefer not to answer	7 (0.5)
Don't know	3 (0.2)
Education	
Less than high school diploma	9 (0.6)
GED or high school diploma	168 (11.6)
Some college, no degree	402 (27.7)
Associate's degree (2-y degree)	224 (15.4)
Bachelor's degree/college degree	399 (27.5)
Beyond college education	250 (17.2)
Prefer not to answer	1 (0.1)
Employment status	
Working full time	694 (47.8)
Working part time	209 (14.4)
Unemployed or laid off	176 (12.1)
Looking for work	49 (3.4)
Keeping house or raising children full time	110 (7.6)
Retired	103 (7.1)
Other	112 (7.7)
Income	
Less than $5,000	45 (3.1)
$5,000-$11,999	67 (4.6)
$12,000-$15,999	54 (3.7)
$16,000-$24,999	148 (10.2)
$25,000-$34,999	211 (14.5)
$35,000-$49,999	224 (15.4)
$50,000-$74,999	310 (21.3)
$75,000-$99,999	177 (12.2)
$100,000 and greater	201 (13.8)
Prefer not to answer	8 (0.5)
Don't know	7 (0.5)
Marital status	
Married	663 (45.7)
Divorced	198 (13.6)
Separated	29 (2.0)
Widowed	32 (2.2)
Single	517 (35.6)
Prefer not to answer	13 (0.9)

GED, general educational development.

### 3.2. Descriptive statistics

Means and SDs of TAS-20 and PAQ total and subscale scores are reported in Supplemental Material A (available at http://links.lww.com/PR9/A258). There was a significant group difference based on racialized identity for TAS-20 total score at T2 (*F* = 4.00, *P* = 0.008). Toronto Alexithymia Scale-20 Item scores were lowest for those who identified as White (mean = 47.70, SD = 12.09), next lowest for Black or African American (mean = 51.64, SD = 11.76), next lowest for the “other” race category (mean = 51.90, SD = 15.02), and finally Asian or Asian American (mean = 52.79, SD = 10.94). The were no group differences based on gender (*F* = 0.571, *P* = 0.565) or education (*F* = 1.41, *P* = 0.196). There were significant negative correlations between TAS-20 and age (*r* = −0.179, *P* < 0.001). In PAQ, there were no group differences based on racialized identity (*F* = 2.506, *P* = 0.058), gender (*F* = 2.45, *P* = 0.87), or education (*F* = 0.169, *P* = 0.991) at T2. There were significant negative correlations between PAQ and age (*r* = −0.144, *P* < 0.001).

### 3.3. Factor structure

Compared to a single factor model, the original 3-factor model showed better model fit in all indices at T1, T2, and T3, indicating that the TAS-20 measured a multidimensional construct. In the original 3-factor model, RMSEAs ranged from 0.082 to 0.083, CFIs ranged from 0.851 to 0.858, and SRMR ranged from 0.076 to 0.081. According to RMSEA, the 3-factor correlated model exhibited marginal levels of model fit in all time points. Comparative fit index indicated poor levels of model fit in all time points. Standardized root mean residual indicated acceptable levels of model fit in T1 and T2, but a marginal level of model fit in T3. Thus, overall, model fit of the TAS-20 3-factor correlated model was deemed to be inadequate. Inspection of factor loadings (Table 2) revealed that the majority EOT items had poor factor loadings (<0.40). See Table 3 for CFA model fit values.

**Table 2:** Standardized item factor loadings from confirmatory factor analyses of the Toronto Alexithymia Scale 20 items in T1, T2, and T3.

Factor/item	T1	T2	T3
DIF			
1. I am often confused about what emotion I am feeling	0.815	0.849	0.842
3. I have physical sensations that even doctors don't understand	0.475	0.464	0.447
6. When I am upset‚ I don't know if I am sad‚ frightened‚ or angry	0.766	0.780	0.793
7. I am often puzzled by sensations in my body	0.604	0.588	0.621
9. I have feelings that I can't quite identify	0.840	0.818	0.866
13. I don't know what's going on inside me	0.780	0.721	0.744
14. I often don't know why I am angry	0.706	0.689	0.693
DDF			
2. It is difficult for me to find the right words for my feelings	0.833	0.868	0.842
4. I am able to describe my feelings easily (R)	0.594	0.607	0.617
11. I find it hard to describe how I feel about people	0.770	0.777	0.759
12. People tell me to describe my feelings more	0.638	0.604	0.642
17. It's difficult for me to reveal my innermost feelings‚ even to close friends	0.530	0.576	0.567
EOT			
5. I prefer to analyze problems rather than just describe them (R)	0.221	0.335	0.208
8. I prefer to just let things happen rather than to understand why they turned out that way	0.328	0.316	0.310
10. Being in touch with emotions is essential (R)	0.669	0.767	0.753
15. I prefer talking to people about their daily activities rather than their feelings	0.364	0.294	0.427
16. I prefer to watch “light” entertainment shows rather than psychological dramas	0.214	0.130	0.266
18. I can feel close to someone‚ even in moments of silence (R)	0.452	0.455	0.417
19. I find examination of my feelings useful in solving personal problems (R)	0.664	0.768	0.751
20. Looking for hidden meanings in movies or plays distracts from their enjoyment	0.276	0.250	0.264

DDF, difficulty describing feelings; DIF, difficulty identifying feelings; EOT, externally oriented thinking; R, reverse coded item.

**Table 3:** Model fit values for the confirmatory factor analysis.

Model	χ^2^ (*df*)	RMSEA (90% CI)	CFI	SRMR
TAS-20 at T1 (single factor model)	2567.510 (170)	0.099 (0.095-0.102)	0.783	0.077
TAS-20 at T1 (3-factor model)	1812.438 (167)	0.082 (0.079-0.086)	0.851	0.076
TAS-20 at T2 (single factor model)	1860.447 (170)	0.107 (0.102-0.111)	0.753	0.086
TAS-20 at T2 (3-factor model)	1169.146 (167)	0.083 (0.079-0.088)	0.854	0.076
TAS-20 at T3 (single factor model)	1765.478 (170)	0.107 (0.103-0.112)	0.755	0.088
TAS-20 at T3 (3-factor model)	1089.438 (167)	0.082 (0.078-0.087)	0.858	0.081
PAQ at T2 (single factor model)	4922.047 (252)	0.147 (0.143-0.151)	0.729	0.098
PAQ at T2 (5-factor model)	1072.079 (242)	0.063 (0.059-0.067)	0.952	0.043
PAQ at T3 (single factor model)	5004.674 (252)	0.152 (0.149-0.156)	0.730	0.100
PAQ at T3 (5-factor model)	1216.546 (242)	0.070 (0.066-0.074)	0.945	0.042

For all examined models, χ^2^ results indicated *P* < 0.001.

CFI, comparative fit index; CI, confidence interval; PAQ, Perth Alexithymia Questionnaire; RMSEA, root mean square error of approximation; SRMR, standardized root mean residual; TAS-20, Toronto Alexithymia Scale 20-item.

Compared to a single factor model, the original 5-factor models showed better model fit at T2 and T3, indicating PAQ measured a multidimensional construct. In the original 5-factor model, RMSEA was 0.063 at T2 and 0.070 at T3, CFI was 0.952 at T2 and 0.945 at T3, and SRMR was 0.043 at T2 and 0.042 at T3. Thus, all of the model fit indices indicated adequate model fit of the PAQ 5-factor model. Inspection of factor loadings (Table 4) revealed that all factor loadings were acceptable in loading well (>0.40) on their intended factor.

**Table 4:** Standardized item factor loadings from confirmatory factor analyses of the 24 Perth Alexithymia Questionnaire items in T2 and T3.

Factor/item	T2	T3
N-DIF		
2. When I'm feeling bad, I can't tell whether I'm sad, angry, or scared	0.825	0.826
8. When I'm feeling bad, I can't make sense of those feelings	0.865	0.896
14. When I'm feeling bad, I get confused about what emotion it is	0.891	0.887
20. When I'm feeling bad, I'm puzzled by those feelings	0.837	0.858
P-DIF		
5. When I'm feeling good, I can't tell whether I'm happy, excited, or amused	0.798	0.849
11. When I'm feeling good, I can't make sense of those feelings	0.873	0.875
17. When I'm feeling good, I get confused about what emotion it is	0.887	0.906
23. When I'm feeling good, I'm puzzled by those feelings	0.867	0.850
N-DDF		
1. When I'm feeling bad (feeling an unpleasant emotion), I can't find the right words to describe those feelings	0.827	0.847
7. When I'm feeling bad, I can't talk about those feelings in much depth or detail	0.749	0.757
13. When something bad happens, it's hard for me to put into words how I'm feeling	0.911	0.887
19. When I'm feeling bad, if I try to describe how I'm feeling I don't know what to say	0.884	0.895
P-DDF		
4. When I'm feeling good (feeling a pleasant emotion), I can't find the right words to describe those feelings	0.814	0.815
10. When I'm feeling good, I can't talk about those feelings in much depth or detail	0.781	0.801
16. When something good happens, it's hard for me to put into words how I'm feeling	0.884	0.901
22. When I'm feeling good, if I try to describe how I'm feeling I don't know what to say	0.867	0.865
G-EOT		
3. I tend to ignore how I feel	0.771	0.776
6. I prefer to just let my feelings happen in the background, rather than focus on them	0.693	0.747
9. I don't pay attention to my emotions	0.802	0.779
12. Usually, I try to avoid thinking about what I'm feeling	0.821	0.847
15. I prefer to focus on things I can actually see or touch, rather than my emotions	0.699	0.751
18. I don't try to be “in touch” with my emotions	0.772	0.756
21. It's not important for me to know what I'm feeling	0.530	0.531
24. It's strange for me to think about my emotions	0.806	0.811

G-EOT, General-Externally Orientated Thinking; N-DDF, Negative-Difficulty Describing Feelings; N-DIF, Negative-Difficulty Identifying Feelings; P-DDF, Positive-Difficulty Describing Feelings; P-DIF, Positive-Difficulty Identifying Feelings.

### 3.4. Reliability

#### 3.4.1. Internal consistency

For TAS-20 total, internal consistency was good. For TAS-20 subscales, internal consistency was good for DIF and DDF, and unacceptably low for EOT. For PAQ, internal consistency was excellent for PAQ total and all subscale scores (Supplemental Table B, available at http://links.lww.com/PR9/A258).

#### 3.4.2. Temporal stability

For both TAS-20 and PAQ, total and subscale scores were highly correlated and large in effect across all time points (Supplemental Table C, available at http://links.lww.com/PR9/A258).

### 3.5. Validity

#### 3.5.1. Construct validity

Regarding divergent validity, TAS-20 and PAQ moderately correlated with PROMIS depression and anxiety. Regarding convergent validity, TAS-20 and PAQ were highly correlated with DERS clarity subscale. Correlations with the DERS total score were relatively less strongly correlated but were still highly correlated and strong in effect. Regarding criterion validity, the correlations between TAS-20 total score and PAQ total score were large in effect (Table 5).

**Table 5:** Summary of criterion, divergent, and convergent validity.

	Criterion validity										Divergent validity		Convergent validity	
	TAS-20				PAQ									
	Total	DIF	DDF	EOT	Total	N-DIF	P-DIF	N-DDF	P-DDF	G-EOT	Depression	Anxiety	DERS: clarity	DERS: total
Total		0.898	0.887	0.630	0.788	0.764	0.632	0.754	0.658	0.591	0.444	0.435	0.720	0.574
DIF			0.815	0.285	0.704	0.787	0.633	0.689	0.617	0.415	0.512	0.523	0.750	0.641
DDF				0.330	0.738	0.707	0.574	0.765	0.649	0.515	0.434	0.433	0.675	0.566
EOT					0.465	0.317	0.299	0.370	0.320	0.539	0.087	0.048	0.280	0.139
Total						0.863	0.831	0.881	0.872	0.820	0.339	0.318	0.699	0.517
N-DIF							0.773	0.821	0.710	0.528	0.396	0.420	0.766	0.613
P-DIF								0.628	0.826	0.506	0.353	0.366	0.634	0.506
N-DDF									0.755	0.614	0.354	0.325	0.678	0.533
P-DDF										0.567	0.352	0.317	0.588	0.459
G-EOT											0.124	0.085	0.433	0.245
Depression												0.759	0.445	0.683
Anxiety													0.460	0.663
DERS: clarity														0.679
DERS: total														

DDF, difficulty describing feelings; DERS, Difficulties in Emotion Regulation Scale; DIF, difficulty identifying feelings; EOT, externally oriented thinking; N, negative; P, positive; PAQ, Perth Alexithymia Questionnaire; TAS-20, Toronto Alexithymia Scale 20-item.

#### 3.5.2. Predictive validity

First, we tested the predictive validity of TAS-20 and PAQ total scores at T2 on pain outcomes at T3. Toronto Alexithymia Scale-20 Item at T2 was significantly associated with pain severity at T3 (controlling for T2 pain severity; B = 0.010, SE = 0.005, *P* = 0.023). Toronto Alexithymia Scale-20 Item at T2 was significantly associated with pain interference at T3 (controlling for T2 pain interference; B = 0.015, SE = 0.007, *P* = 0.029). The relationships between PAQ at T2 and pain severity (B = 0.003, SE = 0.002, *P* = 0.098) and pain interference (B = 0.005, SE = 0.003, *P* = 0.060) at T3 (controlling for T2 pain severity and T2 pain interference respectively) were trending significant.

Next, we conducted a series of sensitivity analyses controlling for symptoms of depression and anxiety at T2. The relationship between TAS-20 total at T2 and pain severity (B = 0.008, SE = 0.005, *P* = 0.132) and pain interference (B = 0.008, SE = 0.007, *P* = 0.292) at T3 became nonsignificant. The relationship between PAQ total at T2 and pain severity (B = 0.002, SE = 0.002, *P* = 0.317) and pain interference (B = 0.003, SE = 0.003, *P* = 0.321) at T3 became nonsignificant.

Finally, we conducted a series of sensitivity analyses after removing 3 somatic-focused TAS-20 items (“I have physical sensations that even doctors don't understand” [item 3], “I am often puzzled by sensations in my body” [item 7], and “I don't know what's going on inside me” [item 13]). The associations between TAS-17 at T2 and pain severity (B = 0.010, SE = 0.005, *P* = 0.076) and pain interference (B = 0.014, SE = 0.008, *P* = 0.077) were trending significant. To further explore this finding, we examined correlations between individual TAS-20 items with pain severity and pain interference. Among all items, the 3 somatic-focused items were most strongly associated with pain severity (*r*s = 0.14-0.34) and pain interference (*r*s = 0.20-0.38) (Supplemental Tables D and E, available at http://links.lww.com/PR9/A258).

## 4. Discussion

The present study tested the psychometric properties of the TAS-20 and PAQ in a large sample of adults with chronic pain. Both measures had psychometric strengths with respect to reliability and validity. Weaknesses included unacceptable internal consistency of the TAS-20 EOT subscale and a failure to replicate the original 3-factor structure of the TAS-20. After accounting for depression and anxiety, neither TAS-20 nor PAQ total scores predicted pain outcomes longitudinally, and we identified concern that somatic-focused items within the TAS-20 may slightly inflate the predictive validity of TAS-20 on pain outcomes.

Replicating most of prior studies, the internal consistency for the TAS-20 total score and its DIF and DDF subscales were good, but the internal consistency of the EOT subscale was unacceptably low.^13,26,39^ Similar to prior studies in clinical samples, we could not replicate the original 3-factor structure of the TAS-20, as most EOT items had poor loadings and the overall model fit did not reach the adequate cutoffs.^22,25,32,52^ The internal consistency of the PAQ was excellent at the total scale and subscale level. The original 5-factor structure of the PAQ had adequate model fit, and all factor loadings were acceptable. More research is needed testing the psychometric properties of the TAS-20 and PAQ in chronic pain; however, these initial findings suggest that the PAQ may be advantageous for researchers interested in interpreting alexithymia subscales.

The TAS-20 and PAQ demonstrated good criterion validity, temporal stability, and convergent validity with emotion regulation. Several studies have found that associations between TAS-20 and pain outcomes were no longer significant after controlling for distress, raising concern that TAS-20 may have poor discriminant validity with psychological distress.^1^ In the current study, we identified no concern for poor convergent validity among TAS-20 and PAQ total scores, although strong correlations between TAS-20 DIF subscale with depression and anxiety does raise the possibility of convergence. The TAS-20 DIF subscale contains somatic items that may conceptually confound with the experience of chronic pain (items 3, 7, 13). Our sensitivity analysis revealed that these items were the most strongly correlated with pain severity and pain interference; however, there was no immediate concern for poor discriminant validity, as correlations were small to moderate in effect.

Longitudinal relationships between TAS-20 and PAQ total scores with pain severity and pain interference were not significant after controlling for depression and anxiety symptoms. The relationship between TAS-20 and pain outcomes weakened after removing potentially confounding items from TAS-20. A dearth of studies parsing sequential relationships between alexithymia, psychological distress, and pain limits interpretation of this finding. For example, alexithymia may lead to increased psychological distress via reductions in emotion regulation, resulting in increased pain.^31^ Pain could lead to increased alexithymia via reduced access to important emotional cues, resulting in depression and anxiety.^1^ Mediation models may help explain the role of psychological distress in the relationship between alexithymia and pain-related outcomes. However, it could also be the case that alexithymia, whether assessed with the TAS-20 or PAQ, does not add incremental validity to measures of psychological distress^30^ in predicting pain-related outcomes. More research is needed to elucidate the—possibly complex—relationship between alexithymia and pain. The use of well-validated alexithymia tools will be essential for this work.

### 4.1. Future research

More research is needed to determine the psychometric properties of the TAS-20 and PAQ in people chronic pain, including determining cross-cultural validity, validity in youth, and measurement invariance. Regarding the latter, we took a preliminary step by testing group differences in TAS-20 and PAQ scores by sociodemographic factors and found that younger age was associated with higher total scores on both TAS-20 and PAQ. Overall, the PAQ demonstrated good psychometric properties and appears to be a good option for studying alexithymia in people with chronic pain, particularly if analysis at both the subscale and total score level is of interest. However, additional research is needed, as this is the first study to our knowledge to use the PAQ in people with chronic pain. Our data suggest that caution should be used when interpreting TAS-20 subscale scores in people with chronic pain. When reporting the total TAS-20 score, investigators might consider reporting sensitivity analyses with the 3 somatic-focused items (3, 7, 13) removed. Although we did not identify major concern for convergence, these items were relatively strongly correlated with pain severity and might slightly inflate the predictive validity of TAS-20 on pain outcomes.

### 4.2. Limitations

Self-reported measures used in the current study can be influenced by response bias and retrospective recall, and there is a need to examine additional measures of emotional awareness and alexithymia in chronic pain, such as performance-based tasks^28^ and interviews.^7^ There is some ongoing debate in the field about whether difficulty daydreaming is a component of alexithymia,^49^ which was not assessed in this study and may be of interest to other researchers. Our sample was primarily White and non-Hispanic, and attrition was higher among participants who were younger, male, Hispanic, and who had lower educational levels. Future studies should take steps to ensure greater representation of minoritized racial and ethnic groups, particularly to formally study measurement invariance.

### 4.3. Conclusion

Both the TAS-20 and PAQ had psychometric strengths in a sample with chronic pain. Total scores of both TAS-20 and PAQ had good internal consistency, construct validity, and temporal validity. Our data indicated several concerns about the TAS-20 in people with chronic pain, including poor factor fit and internal consistency of the EOT subscale, and 3 somatic-focused items on the DIF subscale that might slightly inflate associations between the TAS-20 and pain-related outcomes. For investigators interested in examining alexithymia in chronic pain, our data indicate that the PAQ may be a psychometrically stronger option, although replication is needed. Our findings provide a psychometric foundation upon which to base alexithymia assessment decisions in people with chronic pain.

## Disclosures

Co-author D.A. Preece was an original developer of the Perth Alexithymia Questionnaire. The authors have no other conflicts of interest to disclose.

## Supplemental digital content

Supplemental digital content associated with this article can be found online at http://links.lww.com/PR9/A258.

## Supplementary Material

SUPPLEMENTARY MATERIAL

## References

[1] Aaron RV Fisher E de la Vega R Lumley MA Palermo TM. Alexithymia in individuals with chronic pain and its relation to pain intensity, physical interference, depression and anxiety: a systematic review and meta-analysis. PAIN 2019;160:994–1006. 31009416 10.1097/j.pain.0000000000001487 PMC6688175

[2] Aaron RV McGill LS Finan PH Wegener ST Campbell CM Mun CJ. Determining profiles of pain-specific and general emotion regulation skills and their relation to 12-month outcomes among people with chronic pain. J Pain 2023;24:667–78. 36503109 10.1016/j.jpain.2022.12.002 PMC10079591

[3] Aaron RV Mun CJ McGill LS Finan PH Campbell CM. The longitudinal relationship between emotion regulation and pain-related outcomes: results from a large, online prospective study. J Pain 2022;23:981–94. 34974172 10.1016/j.jpain.2021.12.008 PMC9232929

[4] Asmundson GJ Katz J. Understanding the co-occurrence of anxiety disorders and chronic pain: state-of-the-art. Depress Anxiety 2009;26:888–901. 19691031 10.1002/da.20600

[5] Bagby RM Parker JD Taylor GJ. The twenty-item Toronto Alexithymia Scale—I. Item selection and cross-validation of the factor structure. J Psychosom Res 1994;38:23–32. 8126686 10.1016/0022-3999(94)90005-1

[6] Bagby RM Parker JD Taylor GJ. Twenty-five years with the 20-item Toronto Alexithymia Scale. J Psychosom Res 2020;131:109940. 32007790 10.1016/j.jpsychores.2020.109940

[7] Bagby RM Taylor GJ Parker JD Dickens SE. The development of the Toronto Structured Interview for Alexithymia: item selection, factor structure, reliability and concurrent validity. Psychother Psychosom 2006;75:25–39. 16361872 10.1159/000089224

[8] Bagby RM Taylor GJ Quilty LC Parker JD. Reexamining the factor structure of the 20-item Toronto alexithymia scale: commentary on Gignac, Palmer, and Stough. J Pers Assess 2007;89:258–64. 18001226 10.1080/00223890701629771

[9] Bjureberg J Ljótsson B Tull MT Hedman E Sahlin H Lundh L-G Bjärehed J DiLillo D Messman-Moore T Gumpert CH Gratz KL. Development and validation of a brief version of the difficulties in emotion regulation scale: the DERS-16. J Psychopathol Behav Assess 2016;38:284–96. 27239096 10.1007/s10862-015-9514-x PMC4882111

[10] Cella D Choi SW Condon DM Schalet B Hays RD Rothrock NE Yount S Cook KF Gershon RC Amtmann D DeWalt DA Pilkonis PA Stone AA Weinfurt K Reeve BB. PROMIS adult health profiles: efficient short-form measures of seven health domains. Value Health 2019;22:537–44. 31104731 10.1016/j.jval.2019.02.004 PMC7201383

[11] Chan J Becerra R Weinborn M Preece D. Assessing alexithymia across Asian and Western cultures: psychometric properties of the Perth Alexithymia Questionnaire and Toronto Alexithymia Scale-20 in Singaporean and Australian samples. J Pers Assess 2023;105:396–412. 35900047 10.1080/00223891.2022.2095641

[12] Cleeland CS Ryan KM. Pain assessment: global use of the Brief Pain Inventory. Ann Acad Med Singap 1994;23:129–38. 8080219

[13] Dere J Tang Q Zhu X Cai L Yao S Ryder AG. The cultural shaping of alexithymia: values and externally oriented thinking in a Chinese clinical sample. Compr Psychiatry 2013;54:362–8. 23261082 10.1016/j.comppsych.2012.10.013

[14] DeVellis RF Thorpe CT. Scale development: theory and applications. Thousand Oaks: Sage Publications Inc, 2021.

[15] Fournier A Luminet O Dambrun M Dutheil F Pellissier S Mondillon L. Importance of considering interoceptive abilities in alexithymia assessment. PeerJ 2019;7:e7615. 31768300 10.7717/peerj.7615 PMC6874858

[16] Fredrickson BL. The role of positive emotions in positive psychology: the broaden-and-build theory of positive emotions. Am Psychol 2001;56:218–26. 11315248 10.1037//0003-066x.56.3.218 PMC3122271

[17] Fynn DM Preece DA Gignac GE Pestell CF Allan A Kraats CV Green SL Weinborn M Becerra R. Assessing alexithymia in adults with acquired brain injury: psychometric properties of the Perth Alexithymia Questionnaire. J Affect Disord 2022;302:224–33. 35092756 10.1016/j.jad.2022.01.091

[18] Gignac GE Palmer BR Stough C. A confirmatory factor analytic investigation of the TAS–20: corroboration of a five-factor model and suggestions for improvement. J Pers Assess 2007;89:247–57. 18001225 10.1080/00223890701629730

[19] Gratz KL Roemer L. Multidimensional assessment of emotion regulation and dysregulation: development, factor structure, and initial validation of the difficulties in emotion regulation scale. J Psychopathol Behav Assess 2004;26:41–54.

[20] Gross J. Emotion regulation: current status and future prospects. Psychol Inq 2015;26:1–26.

[21] Groth-Marnat G. Handbook of psychological assessment. Hoboken: John Wiley & Sons, 2009.

[22] Haviland MG Reise SP. Structure of the twenty-item Toronto Alexithymia Scale. J Pers Assess 1996;66:116–25. 8576826 10.1207/s15327752jpa6601_9

[23] Hu L Bentler PM. Cutoff criteria for fit indexes in covariance structure analysis: conventional criteria versus new alternatives. Struct Equat Model 1999;6:1–55.

[24] Keller S Bann CM Dodd SL Schein J Mendoza TR Cleeland CS. Validity of the brief pain inventory for use in documenting the outcomes of patients with noncancer pain. Clin J Pain 2004;20:309–18. 15322437 10.1097/00002508-200409000-00005

[25] Koch AS Kleiman A Wegener I Zur B Imbierowicz K Geiser F Conrad R. Factorial structure of the 20-item Toronto Alexithymia Scale in a large sample of somatoform patients. Psychiatry Res 2015;225:355–63. 25613660 10.1016/j.psychres.2014.12.013

[26] Kooiman C Spinhoven P Trijsburg R. The assessment of alexithymia: a critical review of the literature and a psychometric study of the Toronto Alexithymia Scale-20. J Psychosom Res 2002;53:1083–90. 12479990 10.1016/s0022-3999(02)00348-3

[27] Kroenke K Yu Z Wu J Kean J Monahan PO. Operating characteristics of PROMIS four-item depression and anxiety scales in primary care patients with chronic pain. Pain Med 2014;15:1892–901. 25138978 10.1111/pme.12537 PMC6283354

[28] Lane RD Quinlan DM Schwartz GE Walker PA Zeitlin SB. The levels of Emotional Awareness Scale: a cognitive-developmental measure of emotion. J Pers Assess 1990;55:124–34. 2231235 10.1080/00223891.1990.9674052

[29] Lashkari A Dehghani M Sadeghi-Firoozabadi V Heidari M Khatibi A. Further support for the psychometric properties of the Farsi Version of Perth Alexithymia Questionnaire. Front Psychol 2021;12:657660. 33935916 10.3389/fpsyg.2021.657660 PMC8079730

[30] Leising D Grande T Faber R. The Toronto Alexithymia Scale (TAS-20): a measure of general psychological distress. J Res Personal 2009;43:707–10.

[31] Lumley MA Krohner S Marshall LM Kitts TC Schubiner H Yarns BC. Emotional awareness and other emotional processes: implications for the assessment and treatment of chronic pain. Pain Manag 2021;11:325–32. 33533272 10.2217/pmt-2020-0081 PMC7923252

[32] Müller J Bühner M Ellgring H. Is there a reliable factorial structure in the 20-item Toronto Alexithymia Scale? A comparison of factor models in clinical and normal adult samples. J Psychosom Res 2003;55:561–8. 14642988 10.1016/s0022-3999(03)00033-3

[33] Müller R Segerer W Ronca E Gemperli A Stirnimann D Scheel-Sailer A Jensen MP. Inducing positive emotions to reduce chronic pain: a randomized controlled trial of positive psychology exercises. Disabil Rehabil 2022;44:2691–704. 33264568 10.1080/09638288.2020.1850888

[34] Mun CJ Campbell CM McGill LS Aaron RV. The early impact of COVID-19 on chronic pain: a cross-sectional investigation of a large online sample of individuals with chronic pain in the United States, April to May, 2020. Pain Med 2021;22:470–80. 33537764 10.1093/pm/pnaa446 PMC7901854

[35] Mun CJ Campbell CM McGill LS Wegener ST Aaron RV. Trajectories and individual differences in pain, emotional distress, and prescription opioid misuse during the COVID-19 pandemic: a one-year longitudinal study. J Pain 2022;23:1234–44. 35272053 10.1016/j.jpain.2022.02.005 PMC8898783

[36] Muthén L Muthén B. Mplus (version 8) (1998-2017) [computer software]. Los Angeles: Muthén & Muthén, 2017.

[37] Nahin RL. Estimates of pain prevalence and severity in adults: United States, 2012. J Pain 2015;16:769–80. 26028573 10.1016/j.jpain.2015.05.002 PMC4562413

[38] Preece D Becerra R Robinson K Dandy J Allan A. The psychometric assessment of alexithymia: development and validation of the Perth Alexithymia Questionnaire. Personal Individ Diff 2018;132:32–44.

[39] Preece DA Becerra R Allan A Robinson K Chen W Hasking P Gross JJ. Assessing alexithymia: psychometric properties of the Perth Alexithymia Questionnaire and 20-item Toronto Alexithymia Scale in United States adults. Personal Individ Diff 2020;166:110138.

[40] Preece DA Becerra R Boyes ME Northcott C McGillivray L Hasking PA. Do self-report measures of alexithymia measure alexithymia or general psychological distress? A factor analytic examination across five samples. Personal Individ Diff 2020;155:109721.

[41] Preece DA Gross JJ. Conceptualizing alexithymia. Personal Individ Diff 2023;215:112375.

[42] Preece DA Mehta A Becerra R Chen W Allan A Robinson K Boyes M Hasking P Gross JJ. Why is alexithymia a risk factor for affective disorder symptoms? The role of emotion regulation. J Affect Disord 2022;296:337–41. 34606815 10.1016/j.jad.2021.09.085

[43] Preece DA Mehta A Petrova K Sikka P Pemberton E Gross JJ. Alexithymia profiles and depression, anxiety, and stress. J Affect Disord 2024;357:116–25. 38387670 10.1016/j.jad.2024.02.071

[44] Rayner L Hotopf M Petkova H Matcham F Simpson A McCracken LM. Depression in patients with chronic pain attending a specialised pain treatment centre: prevalence and impact on health care costs. PAIN 2016;157:1472–9. 26963849 10.1097/j.pain.0000000000000542 PMC4912238

[45] Sifneos PE. The prevalence of ‘alexithymic’ characteristics in psychosomatic patients. Psychother Psychosom 1973;22:255–62. 4770536 10.1159/000286529

[46] Stevens JP. Exploratory and confirmatory factor analysis. In: Applied multivariate statistics for the social sciences. New York: Routledge, 2012; 337–406.

[47] Sturgeon JA Zautra AJ. Resilience: a new paradigm for adaptation to chronic pain. Curr Pain Headache Rep 2010;14:105–12. 20425199 10.1007/s11916-010-0095-9 PMC4899321

[48] Tan G Jensen MP Thornby JI Shanti BF. Validation of the Brief Pain Inventory for chronic nonmalignant pain. J Pain 2004;5:133–7. 15042521 10.1016/j.jpain.2003.12.005

[49] Taylor GJ Bagby RM. Examining proposed changes to the conceptualization of the alexithymia construct: the way forward tilts to the past. Psychother Psychosom 2021;90:145–55. 33285546 10.1159/000511988

[50] Taylor GJ Bagby RM Parker JD. The 20-Item Toronto Alexithymia Scale: IV. Reliability and factorial validity in different languages and cultures. J Psychosom Res 2003;55:277–83. 12932803 10.1016/s0022-3999(02)00601-3

[51] Tenti M Raffaeli W Gremigni P. A narrative review of the assessment of depression in chronic pain. Pain Manag Nurs 2022;23:158–67. 33962870 10.1016/j.pmn.2021.03.009

[52] Thorberg FA Young RM Sullivan KA Lyvers M Hurst C Connor JP Feeney GF. A confirmatory factor analysis of the Toronto Alexithymia Scale (TAS-20) in an alcohol-dependent sample. Psychiatry Res 2010;178:565–7. 20510467 10.1016/j.psychres.2009.09.015

[53] Veirman E Van Ryckeghem DM Verleysen G De Paepe AL Crombez G. What do alexithymia items measure? A discriminant content validity study of the Toronto-Alexithymia-Scale–20. PeerJ 2021;9:e11639. 34249500 10.7717/peerj.11639 PMC8253111

[54] Zahid A Taylor GJ Lau SC Stone S Bagby RM. Examining the incremental validity of the Perth Alexithymia Questionnaire (PAQ) relative to the 20-item Toronto Alexithymia Scale (TAS-20). J Personal Assess 2024;106:242–53. 10.1080/00223891.2023.2201831 37144843

