# Supplementary material for: Assessing alexithymia in chronic pain: psychometric properties of the Toronto Alexithymia Scale-20 and Perth Alexithymia Questionnaire
Source: Pain Rep. 2024 Dec 9;10(1):e1204. doi: 10.1097/PR9.0000000000001204 (PMC11631001; doi:10.1097/PR9.0000000000001204)
Supplement: SUPPLEMENTARY MATERIAL [file painreports-10-e1204-s001.pdf]

Supplemental Material

Supplemental Table A

Means and Standard Deviations of TAS-20 and PAQ Total and Subscale Scores

	T1	T2	T3
	Mean (SD)	Mean (SD)	Mean (SD)
**TAS-20**			
Total	50.16 (12.45)	48.23 (12.16)	48.30 (12.19)
DIF	16.75 (6.47)	15.74 (6.11)	15.65 (6.13)
DDF	13.44 (4.33)	12.70 (4.33)	12.70 (4.36)
EOT	19.98 (4.28)	19.78 (4.50)	19.95 (4.46)
**PAQ**			
Total	-	67.81 (29.79)	67.55 (30.98)
N-DIF	-	10.21 (5.98)	9.99 (6.04)
P-DIF	-	8.95 (5.44)	8.79 (5.59)
N-DDF	-	12.55 (6.62)	12.32 (6.77)
P-DDF	-	10.65 (5.97)	10.58 (6.18)
G-EOT	-	25.45 (11.07)	25.86 (11.71)

Supplemental Table B.

Internal Consistency of TAS-20 and PAQ Across Three Timepoints

	T1		T2		T3	
	Alpha	Omega	Alpha	Omega	Alpha	Omega
**TAS-20**						
Total	0.87	0.88	0.87	0.87	0.87	0.88
DIF	0.88	0.88	0.87	0.87	0.88	0.88
DDF	0.81	0.81	0.82	0.82	0.82	0.82
EOT	0.60	0.57	0.63	0.61	0.64	0.62
**PAQ**						
Total	n/a	n/a	0.96	0.96	0.96	0.96
N-DIF	n/a	n/a	0.92	0.92	0.92	0.93
P-DIF	n/a	n/a	0.92	0.92	0.93	0.93
N-DDF	n/a	n/a	0.91	0.91	0.91	0.91
P-DDF	n/a	n/a	0.90	0.90	0.91	0.91
G-EOT	n/a	n/a	0.90	0.91	0.91	0.91

Supplemental Table C

Temporal Stability of TAS-20 and PAQ

	T2	T3
TAS-20		
T1 Total	0.800	0.775
T1 DIF	0.788	0.729
T1 DDF	0.739	0.707
T1 EOT	0.735	0.734
PAQ		
T2 Total		0.816
T2 N-DIF		0.726
T2 P-DIF		0.750
T2 N-DDF		0.750
T2 P-DDF		0.743
T2 EOT		0.789

Note. Values depict Pearson correlations

Supplemental Table C

Item level correlations between TAS-20 and pain severity and pain interference.

		Pain Severity			Pain Interference		
TAS-20 Item	Scale	T1	T2	T3	T1	T2	T3
Tas-20 10†	EOT	-0.02	-0.06	-0.03	0.03	0.02	-0.04
TAS-20 5†	EOT	-0.02	0.03	0.02	0.00	0.00	-0.01
TAS-20 15	EOT	-0.02	-0.06	-0.01	0.02	0.01	0.01
TAS-20 19†	EOT	0.00	0.01	-0.02	0.04	0.06	-0.01
TAS-20 16	EOT	0.00	-0.01	-0.02	0.07**	0.01	-0.01
TAS-20 8	EOT	0.02	0.04	-0.02	0.05*	0.04	0.00
TAS-20 18†	EOT	0.03	0.10**	0.07*	0.08**	0.11**	0.07*
TAS-20 4†	EOT	0.03	0.01	0.03	0.06*	0.12**	0.10**
TAS-20 20	EOT	0.04	0.06	-0.01	0.09**	0.10**	0.00
TAS-20 12	DDF	0.06*	0.03	0.10**	0.12**	0.10**	0.11**
TAS-20 11	DDF	0.07**	0.06	0.09*	0.16**	0.16**	0.12**
TAS-20 9	DIF	0.08**	0.09**	0.15**	0.18**	0.19**	0.20**
TAS-20 17	DDF	0.09**	0.03	0.07	0.13**	0.17**	0.09*
TAS-20 6	DIF	0.10**	0.10**	0.13**	0.20**	0.16**	0.18**
TAS-20 14	DIF	0.11**	0.11**	0.13**	0.19**	0.22**	0.19**
TAS-20 1	DIF	0.12**	0.14**	0.16**	0.21**	0.23**	0.20**
TAS-20 2	DDF	0.13**	0.12**	0.14**	0.21**	0.24**	0.19**
TAS-20 13	DIF	0.14**	0.14**	0.15**	0.23**	0.26**	0.20**
TAS-20 7	DIF	0.15**	0.19**	0.20**	0.27**	0.29**	0.29**
TAS-20 3	DIF	0.22**	0.30**	0.34**	0.31**	0.36**	0.38**

Note. For ease of interpretation, TAS-20 items are arranged in ascending order by strength of correlations between TAS-20 item and pain severity at T1.

* $p < .05$; ** $p < .01$.

†Indicates reverse scored item.

Supplemental Table D

Item level correlations between PAQ and pain severity and pain interference.

	Scale	Pain Severity		Pain Interference	
		T1	T2	T1	T2
PAQ 15	G-EOT	-0.04	0.00	0.02	0.05
PAQ 9	G-EOT	-0.02	-0.01	0.07	-0.03
PAQ 24	G-EOT	-0.01	0.04	0.07*	0.05
PAQ 21	G-EOT	0.00	0.00	0.03	0.03
PAQ 18	G-EOT	0.00	-0.02	0.03	0.02
PAQ 12	G-EOT	0.03	0.09*	0.14**	0.13**
PAQ 6	G-EOT	0.03	0.01	0.10**	0.03
PAQ 22	P-DDF	0.03	0.05	0.13**	0.09**
PAQ 20	N-DIF	0.04	0.11**	0.13**	0.20**
PAQ 19	N-DDF	0.05	0.07*	0.17**	0.14**
PAQ 3	G-EOT	0.05	0.05	0.11**	0.09**
PAQ 13	N-DDF	0.05	0.09**	0.17**	0.16**
PAQ 8	N-DIF	0.07*	0.13**	0.19**	0.18**
PAQ 11	P-DIF	0.07*	0.08*	0.19**	0.12**
PAQ 5	P-DIF	0.07*	0.11**	0.15**	0.15**
PAQ 14	N-DIF	0.07*	0.09**	0.13**	0.15**
PAQ 7	N-DDF	0.08*	0.11**	0.17**	0.17**
PAQ 17	P-DIF	0.08*	0.07*	0.17**	0.12**
PAQ 16	P-DDF	0.08*	0.08*	0.20**	0.12**
PAQ 23	P-DIF	0.09*	0.08*	0.19**	0.14**
PAQ 10	P-DDF	0.09**	0.08*	0.18**	0.12**
PAQ 2	N-DIF	0.10**	0.13**	0.19**	0.17**
PAQ 4	P-DDF	0.10**	0.07*	0.21**	0.11**
PAQ 1	N-DDF	0.11**	0.13**	0.21**	0.15**

Note. For ease of interpretation, PAQ items are arranged in ascending order by strength of correlations between PAQ item and pain severity at T1.

* $p < .05$; ** $p < .01$.
